# Progress of the State Faculty Bill

**Published:** 1884-01

**Authors:** 


### Editorial.

### Progress of the State Faculty Bill.

As the time for the assembling of our legislature draws near, the interest in the effort to secure the passage of the State Faculty bill naturally increases. Since our last issue, prominent members of the press have given the bill editorial mention, and in the cases of the *Commercial* and *Express* of Buffalo, of the *Albany Evening Journal* and the *Syracuse Standard*, the mention has been quite commendatory.

This is a most encouraging sign and shows, to a demonstration, that the bill is drawn, not in the spirit of faction or in the interest of cliques, but in the interest of the medical profession; and this interest is inseparable from the good of the people.

The time for holding the stated meetings of the county societies is in general later than this, but still the committee having the subject in charge have received official notification of the endorsement of the bill by the societies of Wayne, Westchester, Rockland, Cayuga, Oswego, Chemung, Herkimer and Niagara, and what seems quite remarkable, when the uncompromising character of the measure is considered, in almost every case the endorsement has been by unanimous vote.

In addition to this, letters are received from various points in the State, pledging the best efforts of the best men in the profession to this cause. We have also remarked, with great commendation, the freedom from extravagant expression in the official utterances in this connection. Doubtless, there is in the minds of many a strong feeling adverse to the schools, and yet in every instance that has come to our notice there is a full and ready recognition of all their legitimate rights, and a most remarkable forbearance and moderation in the treatment of this feature of the case. As an instance, we cannot do better than produce, as we are permitted to do, the following account of the action of the medical society of a neighboring county:

ELMIRA, December 20, 1883.

At a meeting of the Medical Society of the County of Chemung, held at Elmira, December 18, 1883, the following preamble and resolution, offered by Dr. Wm. Woodward, delegate to the Medical Society of the State of New York, were unanimously adopted.

F. W. ROSS,

Secretary.

WM. C. WEY,

President.

WHEREAS, The act proposed by the Medical Society of the County of Erie to establish the Medical Faculty of the University of the State of New York, is designed not to restrict the powers of Medical Colleges to confer diplomas, but to create an independent board, with authority to review the qualifications of such graduates, for the purpose of giving or withholding license to practice medicine and surgery in the State, as may be determined by a rigid and impartial examination; and, whereas, the said act to establish the Medical Faculty of the University of the State of New York is approved and recommended by the standing committee on legislation of the Medical Society of the State of New York; it is, therefore,

*Resolved*, That this Society urge upon the senator from the 27th district, the Hon. J. Sloat Fassett, and the member of assembly from the county of Chemung, the Hon. J. S. Van Duzer, to use all laudable efforts, in the approaching session of the legislature, to secure the passage of the act mentioned in the foregoing preamble, which is made necessary in consequence of the great increase of medical graduates, who, in many cases, are insufficiently educated, who become a reproach to the schools which give them authority to practice, and bring discredit to the name and dignity of the profession of medicine.

There is in this calm and dignified statement, that which we believe to be the earnest of a quiet, well considered determination to put the hand to the plow, and not to turn back until the State Faculty is an accomplished fact.

Perhaps of more importance than any other of the events of the last month is the appearance, in the struggle for better methods in the licensing of physicians, of the Committee on Legislation of the Medical Society of the State of New York.

Some of our readers may have wondered why a single county society was permitted to take the initiative in a work of this importance. The reasons for this the State committee now give in a circular letter, which they will soon address to the profession.

This letter is to be sent in connection with the bill, amended in accordance with the various timely suggestions developed by the correspondence incident to its introduction to the profession at large.

We have seen an advance proof of this letter and can say to our readers, that not only has the entire action of the committee of the local society been under the advice of the State committee, but the latter now comes to the front and throws its weight and the influence of the State Medical Society upon the side of the State Faculty bill.

But one more topic, and this case is brought down to date. We hear that the New York medical schools begin to realize that this is more than a passing shower, and that they have prepared a substitute for the State Faculty bill, a copy of which lies before us.

This bill seems worthy of notice for many reasons. Because it will be offered as a substitute for the one which has been before the profession for many months, and everywhere recognized as a measure framed in the interests of the whole profession, the strength of which is made more apparent by comparison with the substitute. Then the measure is of note because its appearance, at the suggestion of the medical schools, shows that the necessity for improvement upon the present methods of licensing physicians is imperative and acknowledged by all, that the only question is, as to how the remedy shall be applied. This proposed substitute is also of interest, because its provisions demonstrate that the habits of thought of its authors, do not permit them to embrace any interests beyond those of the corporations which they are engaged in managing.

The medical college bill follows the State Faculty bill in most of its provisions, making one important exception. In the latter all persons who propose to begin the practice of medicine in the State after November 1, 1884, must be examined and licensed by this faculty. In the substitute, this rule holds for all persons coming from without the State, but for graduates of our medical schools quite another rule obtains, and in this the bill and its alleged substitute differ widely. The medical college bill provides that any medical school in this State, about to hold its annual graduation exercises, shall so intimate to the State Faculty, which body shall at once dispatch three of its members as delegates, who shall attend such exercises; these delegates shall have the privilege of knowing what questions are to be asked the candidates for graduation and of reading their answers to the same, when such shall be in writing. When the exercises are over, they have the privilege of returning home and of reporting to the Regents the names of all such graduates as wish licenses to practice in this State, and upon such report, and the payment of a fee of five dollars in each case, such graduates shall be licensed by the Regents of the University.

How could anything be more considerate of the interests of our schools or more thoughtful of the feelings of our State Faculty? The latter have not even the trouble of asking a single question; they have simply to hear the question and its answer, or to read the same. Perhaps they may be called upon to attend a few faculty dinners in the meantime, and then go home with the sweet satisfaction of having discharged all the duty the law laid upon them.

And this is the way certain schools propose to answer the imperative demand of the profession, for the absolute, unconditional divorce of the teaching and licensing powers. We heartily thank these schools for giving us their whole mind upon the question, but have serious doubts about the substitute being an improvement upon the original; and improvements seem to be the order of the day.

